# Longitudinal Study of Subclinical Mastitis in Sheep in Greece: An Investigation into Incidence Risk, Associations with Milk Quality and Risk Factors of the Infection

**DOI:** 10.3390/ani13203295

**Published:** 2023-10-22

**Authors:** Charalambia K. Michael, Daphne T. Lianou, Natalia G. C. Vasileiou, Vasia S. Mavrogianni, Efthymia Petinaki, George C. Fthenakis

**Affiliations:** 1 Veterinary Faculty, University of Thessaly, 43100 Karditsa, Greece; 2 Faculty of Animal Science, University of Thessaly, 41110 Larissa, Greece; 3 University Hospital of Larissa, 41110 Larissa, Greece

**Keywords:** farmer demography, goat, mastitis, milk, predictor, prevalence, sheep, somatic cell counts, *Staphylococcus*, subclinical mastitis, vaccination

## Abstract

**Simple Summary:**

The work presents a study of subclinical mastitis in dairy sheep in Greece, during which we carried out repeated examinations of mammary secretion samples from individual animals and of milk samples from the bulk tank in the farms. The findings indicate that the risk for subclinical mastitis is over 50% throughout a milking period, with staphylococci being the most important causal agents of the infection. There was a clear correlation between the frequency of subclinical mastitis in the flocks and the quality of bulk-tank milk produced in the farm. Younger age of newborns when taken away from the dam and consequently delayed start of milking of ewes, omission of anti-mastitis vaccination of ewes and lack of employed staff on the farms were found to be associated with a higher incidence of the infection.

**Abstract:**

The objectives of this work were (a) to describe the incidence risk of subclinical mastitis in dairy flocks throughout the milking period, (b) to present potential associations of subclinical mastitis with the quality of milk and (c) to identify risk factors for high-incidence risk of the infection. A longitudinal study was performed in 12 dairy sheep flocks in Greece. Mammary secretion samples from 240 ewes and bulk-tank milk samples were collected in four repeated visits over a period of six months for bacteriological, chemical and cytological examinations. Overall, the incidence risk of subclinical mastitis throughout the study period was 51.7%, and it varied among farms from 25.0% to 75.0%. The respective figure for staphylococcal subclinical mastitis was 48.8%. The incidence risk of recurrence of subclinical mastitis among ewes in the flocks was 35.4%. The most frequently identified bacteria from cases of subclinical mastitis were *S. aureus* and *S. simulans*; of the mastitis-causing staphylococcal isolates, 65.4% were biofilm-forming. Somatic cell counts in bulk-tank milk progressively increased as the lactation period advanced, with significant increases seen on the third and fourth visits to the farms. Somatic cell counts in bulk-tank milk correlated well with prevalence of subclinical mastitis in flocks. A significant inverse correlation and a significant positive correlation were seen for prevalence of subclinical mastitis versus total protein content or added water in bulk-tank milk. During multivariable analysis, younger age of newborns when taken away from the dam and consequently delayed start of milking of ewes, omission of anti-mastitis vaccination of ewes and lack of employed staff on the farms emerged to be significantly associated (positively) with increased incidence risk of subclinical mastitis.

## 1. Introduction

In sheep, mastitis adversely affects production and causes financial problems, especially in dairy farms; it has also been recognized as the most important cause of concern about welfare of ewes [1]. The adverse economic effects of ovine mastitis have been recognized and described [2]. In dairy sheep farms, these refer primarily to the reduced milk production, as well as to the suboptimal milk quality, which results in reduction of the price that milk is purchased at by dairy companies. Rightly, therefore, shepherds have declared mastitis as the most important health problem that might occur in their farms [3].

In several approaches, prevention of ovine mastitis is based on transferring and applying knowledge referring to cattle herds directly to sheep flocks, for example, by applying post-milking teat dipping. Nevertheless, there are significant differences between ewes and cows; these refer to the anatomy and physiology of the mammary gland, to the reproductive patterns of the animals, to the management systems applied in the farms, etc. With specific reference to the application of post-milking teat dipping, it is noteworthy that this is infrequently practiced in dairy sheep, for example, in only 20% of the flocks in France [4] and in 16% of the flocks in Greece [3]; moreover, omission of the practice in dairy flocks has not been found to be associated with high somatic cell counts in the bulk-tank milk of dairy sheep farms [3], indicating that potentially other factors could be more influential in the prevention of the infection.

In Greece, sheep production is the predominant form of agriculture, with over 95% of ewes farmed for dairy production. Hence, there is a scope in studying and monitoring the situation regarding mastitis in sheep farms in the country, as well as in identifying potential risk factors for the infection.

The objectives of the work presented in this paper were (a) the description of the incidence risk of subclinical mastitis in dairy flocks throughout the milking period, (b) the presentation of potential associations of subclinical mastitis with the quality of milk and (c) the identification of risk factors for high-incidence risk of the infection.

## 2. Materials and Methods

### 2.1. Sheep Flocks and Sample Collection

A longitudinal study was performed from November 2019 to July 2020. A total of 12 dairy, machine-milked sheep flocks were included in the study (Table S1) and visited on repeated occasions for examining animals, collecting samples and obtaining information. The flocks were selected by collaborating veterinarians on a convenience basis (willingness of farmers to accept repeated visits by university staff for sample collection). The farms were visited four times during a milking period: the first visit was performed within five days after removal of lambs from the dams and the start of milking of the ewes, whilst the second and the third were performed one and a half months after the preceding visit; the fourth visit was performed three months after the third. Another visit had been planned to take place in between the third and the actual fourth visit, but that coincided with the initial quarantine period during the COVID-19 pandemic, in spring 2020; in Greece, that period lasted for two months, and during that period, all movements of people across the country were limited to the absolute minimum. Within 7 to 10 days after the final visit, the milking period in the flocks ended. That way, a total milking period of six to six and a half months was monitored in the flocks.

Initially, an interview of the farmer was performed by using a detailed questionnaire to record management practices and health issues in the flock [3]. On each farm, 20 ewes were selected for collection of mammary secretion samples by using a standardized protocol, previously described in detail [5]. In brief, this included the random selection of clinically healthy, secundiparae or older ewes, which was performed by means of an electronic random number generator. The animals were tagged (in additional to the standard ear tags born by the animals) for identification in the repeated samplings.

Before sampling, a standardized clinical examination of the udder (observation, palpation, comparison between mammary glands) was performed in each ewe, as described before [5]. As the aim of the work was the study of subclinical mastitis, none of the ewes sampled had any clinically detectable mammary abnormalities, nor any other type of relevant problem. Sample collection was carried out by following aseptic procedures (e.g., handling with gloved hands, teat disinfection, use of sterile containers) according to protocols previously detailed [5]. From each of these 20 ewes, mammary secretion samples were collected from both mammary glands into separate containers from each gland.

Thereafter, four 20 mL samples were collected from the bulk-tank milk of the farm by using aseptic sampling techniques and following protocols previously detailed [3]. All transportation of samples was carried out by the principal investigators. Samples were stored in portable refrigerators with ice packs and transported by car.

### 2.2. Laboratory Examinations

#### 2.2.1. Cytological Examinations

After sample collection from animals, at ewe-side, mammary secretion samples were assessed by means of the California Mastitis Test (CMT). The test was performed as previously described for ewes’ milk [6]. Five degrees of reaction (‘negative’, ‘trace’, ‘l’, ‘2’, ‘3’) were described [7].

Mammary secretion smears were also made from the samples obtained from the individual animals and were dried.

Within 4 h of sample collection from bulk-tank milk, two of the four samples were used for somatic cell counting. Two sub-samples were created from each sample and processed, so that each cell counting was performed four times (each time in a different sub-sample). Somatic cell counting (Lactoscan SCC; Milkotronic Ltd., Nova Zagora, Bulgaria) was performed on each of these four sub-samples within 4 h after sample collection.

The secretion smears previously prepared were stained by the Giemsa method; the percentage (%) of types of leucocytes in there was estimated by counting the leucocytes during the observation of at least 10 fields of each milk film using magnification 10×.

Finally, the Microscopic cell counting method (Mccm) (IDF reference method) [8,9,10] was performed in 393 samples of mammary secretion (0.207 of all samples collected).

#### 2.2.2. Examination for Chemical Composition

Immediately after completion of somatic cell counting, milk composition measurement was performed on each of the same four sub-samples by means of an electronic analyzer (Lactoscan Farm Eco; Milkotronic Ltd., Nova Zagora, Bulgaria)

#### 2.2.3. Microbiological Examinations

Bacteriological examinations started within 24 h after collection of samples.

All mammary secretion samples from individual animals (10 μL) were cultured on Columbia blood agar plates (BioPrepare Microbiology, Athens, Greece) and on staphylococcus selective medium (mannitol salt agar; BioPrepare Microbiology, Athens, Greece), which were incubated aerobically at 37 °C for 48 h. If nothing had grown, media were re-incubated for another 24 h. Bacterial identifications were performed by using standard microbiological methods [11,12].

The remaining two of the four milk samples collected from the bulk tank were used for microbiological examination. Two sub-samples were created from each sample and processed, so that each microbiological procedure (as below) was performed four times (each time in a different sub-sample). The four sub-samples were cultured on staphylococcus selective medium; all plates were incubated aerobically at 37 °C for 48 h; if there was no growth, the plates were re-incubated for another 24 h.

Finally, total bacterial counts were performed on each of these four sub-samples. The procedures described in detail by Laird et al. [13] were followed. After completion of sample aliquot withdrawal for microbiological examination, the temperature of the respective samples was measured and in no case was found to exceed 3.8 °C.

In all cases, bacterial isolation and initial identification were performed using standard methods [11,12]. Staphylococcal isolates obtained from cases of subclinical mastitis or obtained from bulk-tank milk were identified to species level by means of Matrix-Assisted Laser Desorption/Ionization Time-of-Flight Mass Spectrometry (MALDI-TOF MS) (VITEK MS; BioMerieux, Mar-cy-l’-Étoile, France). Finally, in all staphylococcal isolates, independently of their origin, the in vitro formation of biofilm by the isolates was tested by combining the findings of (a) culture appearance on Congo red agar plates and (b) results of the microplate adhesion test, as detailed by Vasileiou [5].

### 2.3. Data Management and Analysis

#### 2.3.1. Data Management

Subclinical mastitis was defined in ewes, in which a bacteriologically positive mammary secretion sample: [a] >10 colonies of the same organism and [b] no more than two different types of colonies, with concurrently increased cell content: [a] CMT score ≥‘1’ and [b] neutrophil and lymphocyte proportion cumulatively ≥ 65% of all leucocytes, was detected, with no presence of abnormal gross findings in the mammary gland (including changes in secretion) [5]. Staphylococcal subclinical mastitis was defined in ewes, in which *Staphylococcus* spp. was identified as a causal agent of subclinical mastitis. Mammary bacterial carriage was considered in ewes, in which a bacteriologically positive milk sample (as detailed hereabove) without concurrently increased cell content: [a] CMT score (≤‘trace’) and/or [b] neutrophil and lymphocyte proportion cumulatively <65% of all leucocytes, was detected; the term mammary bacterial carriage is used to describe presence of bacteria in the mammary gland with no increased somatic cell numbers (i.e., in the absence of inflammation). Clinical mammary abnormalities referred to the presence of abnormal formations in a mammary gland of ewes, as detected by means of physical examination (observation, palpation). Recurrence of subclinical mastitis was noted when cases of subclinical mastitis caused by different pathogens were recorded in the same ewe on different sampling occasions.

All above outcomes referred to ewes. Hence, animals with both glands affected were counted as one case.

Incidence risk for each of the above outcomes was defined as the proportion of animals at risk that developed the condition when the time at risk differed between animals (i.e., during the entire milking period, which varied between individuals) [5]. Animals that cleared the infection (i.e., were found to not have subclinical mastitis) were considered at risk at subsequent visits; if they developed the infection again during the study period, they were still counted as one case.

Quantitative information on the cellular content of ewes’ mammary secretion was obtained by using two sets of data: the CMT results and the results of the Mccm. Although it is well known that CMT results are reliable proxy measurements for somatic cell counts [6,14], this was further assured in the present study. Following assignment of numerical values to CMT scores (value 0 to score ‘negative’, value 1 to score ‘trace’, value 2 to score ‘1’, value 3 to score ‘2’ and value 4 to score ‘3’) and transformation of somatic cell counts to somatic cell scores as defined below, correlation analysis between CMT scores and Mccm SCCs was performed; the correlation coefficient was found to be *r* = 0.840 (95% confidence interval [CI]: 0.809—0.867) (*p* < 0.0001), and the R^2^ was 70.6%.

For the bulk-tank milk samples, detection of at least three confirmed staphylococcal colonies on at least one agar plate of the four plates cultured with each bulk-tank milk sub-sample from each flock was considered to indicate presence of the organism.

For characterization of biofilm formation by staphylococcal isolates, the results of the two methods that had been employed were combined [14], and the isolates were characterized as biofilm-forming or non-biofilm-forming.

For all statistical analyses, SCCs were transformed to somatic cell scores (SCS) as described by Wiggans and Shook [15] and Franzoi et al. [16]: SCS = log_2_(SCC/100) + 3, whilst total bacterial counts were transformed to log_10_, and the transformed data were used in the analyses; then, for presentation of the results, the transformed findings were back-transformed into 100 × 2^(SCS−3)^ and 10^log^ data, respectively.

#### 2.3.2. Statistical Analysis

All data were entered into Microsoft Excel and analyzed using IBM SPSS Statistics (ver. 21) (IBM; Armonk, NY, USA). Basic descriptive analysis was performed. Exact binomial confidence intervals (CI) were obtained.

Frequencies were compared by using Pearson’s chi-square test or Fisher exact test, as appropriate. The differences between the progressive samplings in somatic cell counts, total bacterial counts and chemical composition parameters (fat content, total protein content, added water) in bulk-tank milk of the 12 flocks were assessed by using analysis of covariance. Analysis of correlation between the prevalence of mastitis in each flock on each sampling occasion with somatic cell counts, total bacterial counts and chemical composition parameters in bulk-tank milk on the respective occasion was performed by means of Spearman’s rank correlation.

For the identification of potential risk factors for subclinical mastitis and staphylococcal subclinical mastitis, first, the outcomes ‘incidence risk of subclinical mastitis’ and ‘incidence risk of staphylococcal subclinical mastitis’ were considered. In total, 67 parameters (related to infrastructure, animals, production characteristics, health management and human resources in the flock; Table S2) were evaluated for potential association with these outcomes. For each of these parameters, categories were created according to the replies of the farmers, with results taken directly from the answers obtained during the interview or calculated based on these answers. Initially, in univariable analyses, the importance of predictors was evaluated by using Spearman’s rank correlation of the incidence risk of subclinical mastitis with the results of the various parameters assessed. Then, a multivariable model was developed for the above outcome; parameters found with *p* < 0.2 in the preceding univariable analyses were offered to this model. Progressively, variables offered into the multivariable model were removed from the model by using backwards elimination. The likelihood ratio test was performed to assess the *p*-value of each parameter; among those found with *p* ≥ 0.2, the one with the largest *p* was removed from the model. The procedure was repeated until no variable with *p* ≥ 0.2 could be removed from the model. The variables included in the final multivariable model constructed are detailed in Table S3.

In all analyses, statistical significance was defined at *p* < 0.05.

## 3. Results

### 3.1. Incidence Risk of Subclinical Mastitis

In total, 240 ewes were examined throughout their milking period. Milking had stopped in seven ewes before the end of the study (in all, before the fourth visit to the farms). In total, 948 samplings of ewes with clinically normal udders were performed during the study (i.e., 1896 mammary secretion samples were collected from individual ewes).

Three ewes in three farms were found to have developed clinical mammary abnormalities: in two ewes on the third visit and in the third ewe on the fourth. The incidence risk of clinical mammary abnormalities was 1.3% (95% CI: 0.4–3.6%).

Overall, the incidence risk of subclinical mastitis throughout the study period was 51.7% (95% CI: 45.4–57.9%). The incidence risk varied between farms from 25.0% to 75.0% (*p* = 0.0005) (Table S4). The estimated incidence risk of subclinical mastitis in the study farms combined was 55.2% (95% C.I.: 53.3–57.0%).

The incidence risk throughout the first three months of the milking period was 35.4% (95% CI: 29.6–41.7%); it varied between farms from 10.0% to 55.0% (*p* = 0.07). The incidence risk of subclinical mastitis during the last three months of the milking period was 24.4% (95% CI: 19.3–30.3%) (*p* = 0.009 for comparison between the two stages of the milking period).

Overall, the incidence risk of staphylococcal subclinical mastitis was 48.8% (95% CI: 42.5–55.0%) (Table S4). The incidence risk throughout the first three months of the milking period was 32.9% (95% CI: 27.3–39.1%).

Recurrence of subclinical mastitis was noted in 35 ewes; the incidence risk of recurrence of subclinical mastitis was 35.4% (Table S4). There was a clear correlation between the incidence of subclinical mastitis in a flock and the cases of recurrence of subclinical mastitis recorded in the same flock during the study period (*r_sp_* = 0.653, *p* = 0.021).

Prevalence of subclinical mastitis progressively increased, from 12.5% on the first visit to 26.1% on the fourth (*p* = 0.0007 between sampling occasions). Among all the samplings performed, subclinical mastitis was evident on 187 occasions (19.7%). In most of these cases (176 of 187; 94.1%), staphylococcal subclinical mastitis was detected.

Mammary bacterial carriage was seen on 152 occasions (16.0%) during the study.

### 3.2. Identity of Bacteria Isolated from Mammary Secretion Samples during the Study

The most frequently identified bacterial isolates from cases of subclinical mastitis were *S. aureus* (*n* = 38) and *S. simulans* (*n* = 34) (Table 1). Among isolates from cases of bacterial carriage, non-*aureus* staphylococci were most frequently recovered (*n* = 148, 92.5%) (Table S5).

Most staphylococcal isolates recovered from cases of subclinical mastitis were biofilm-forming (*n* = 117, 65.4%). In contrast, most isolates recovered from cases of mammary carriage were not (*n* = 50, 33.8%) (*p* < 0.0001) (Table S6). The proportion of biofilm-forming isolates among *S. aureus* (84.2%) was significantly higher than that among non-*aureus* isolates (60.3%) (*p* = 0.006).

### 3.3. Findings in Bulk-Tank Milk and Correlations with Prevalence of Subclinical Mastitis

#### 3.3.1. Somatic Cell Counts and Total Bacterial Counts

**Figure 1:**
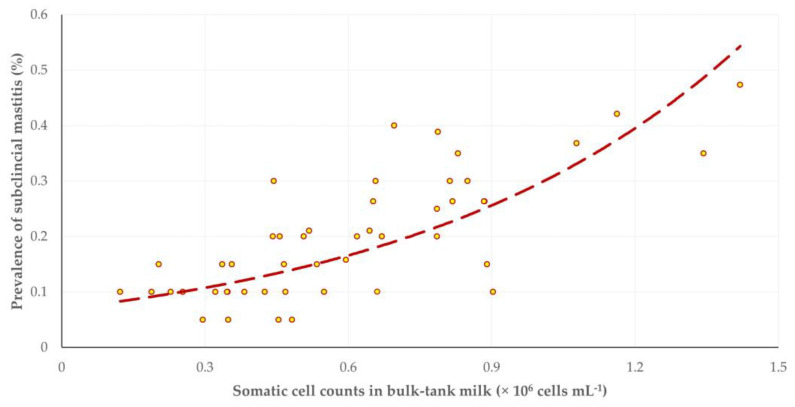
Scatterplot of prevalence of subclinical mastitis and somatic cell counts in bulk-tank milk in 12 sheep flocks in Greece monitored throughout a milking period (dashed line is trendline).

**Figure 2:**
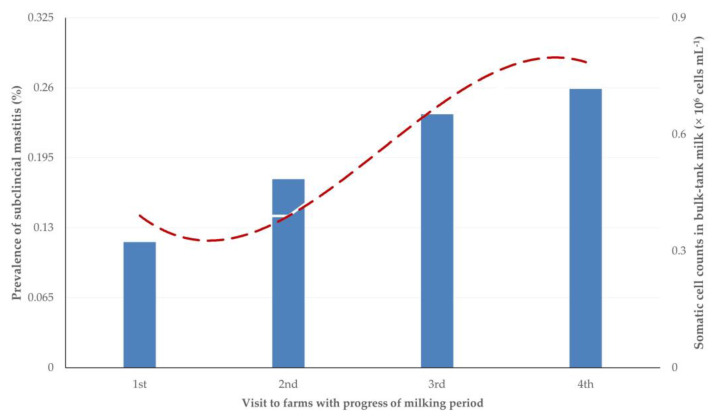
Progressive changes in prevalence of subclinical mastitis and in somatic cell counts in bulk-tank milk in 12 sheep flocks in Greece monitored throughout a milking period (four sampling occasions on each flock).

Somatic cell counts in bulk-tank milk progressively increased as the lactation period advanced, with significant differences seen on the third and fourth visits to the farms (*p* = 0.0005) (Table 2). There was a positive correlation between the prevalence of subclinical mastitis in a flock on a sampling occasion with the respective somatic cell counts in the bulk tank milk (*r_sp_* = 0.711, *p* < 0.0001) (Figure 1); moreover, there was no difference between the regression slopes of subclinical mastitis prevalence (0.023 ± 0.135) and the somatic cell counts in bulk-tank milk (0.073 ± 0.368) during the study (*p* = 0.37) (Figure 2).

Total bacterial counts in bulk-tank milk also progressively increased as the lactation period advanced, with significant differences seen on the fourth visit to the farm (*p* = 0.035) (Table 2). There was a correlation between the prevalence of mastitis in a flock on a sampling occasion with the respective total bacterial counts in the bulk tank milk (*r_sp_* = 0.445, *p* = 0.002) (Figure S1).

There was a tendency for significant difference between the correlation coefficients for the prevalence of subclinical mastitis in a flock on a sampling occasion versus the somatic cell counts or the total bacterial counts in the bulk-tank milk on the same sampling occasion (*z* = –1.95, *p* = 0.051).

#### 3.3.2. Chemical Composition

**Figure 3:**
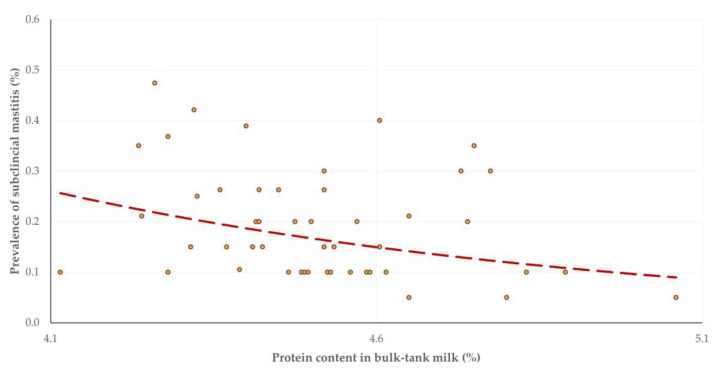
Scatterplot of prevalence of subclinical mastitis and protein content in bulk-tank milk in 12 sheep flocks in Greece monitored throughout a milking period (dashed line is trendline).

**Figure 4:**
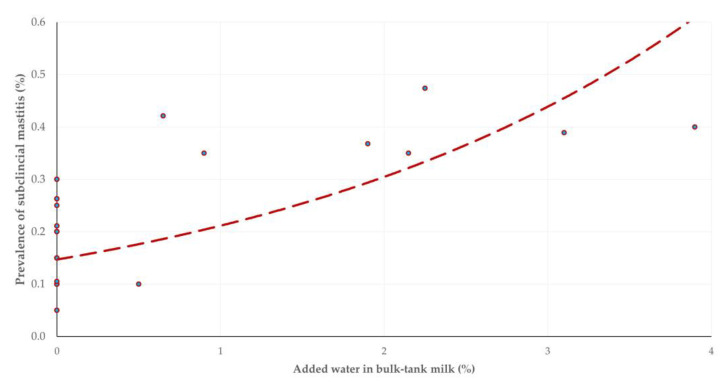
Scatterplot of prevalence of subclinical mastitis and added water in bulk-tank milk in 12 sheep flocks in Greece monitored throughout a milking period (dashed line is trendline).

There was no change in fat content in bulk-tank milk with the advancement of the milking period (*p* = 0.94); in contrast, there was a significant decrease in protein content and a mild increase in added water in there (*p* = 0.004 and *p* = 0.11, respectively) (Table 3). A significant inverse correlation was seen between the prevalence of subclinical mastitis in a flock and the total protein content in the bulk-tank milk (*r_sp_* = –0.325, *p* = 0.024) (Figure 3). Moreover, a positive correlation was found between the prevalence of subclinical mastitis in a flock and the added water in the bulk-tank milk (*r_sp_* = 0.549, *p* = 0.001) (Figure 4).

#### 3.3.3. Identity of Staphylococci Isolated

Staphylococci were isolated from the bulk-tank milk of all 12 farms on 25 sampling occasions in total (52.1%), with a median of isolation on two occasions per farm (min. 1–max. 4 occasions) during the study. There was no significant difference between the sampling occasions in the number of farms from the bulk-tank milk of which staphylococci were isolated (from 33.3% to 75.0% of farms per sampling occasion) (*p* = 0.18).

The most frequently identified species in samples of bulk-tank milk was *S. aureus* (*n* = 9) (Table 4).

Most staphylococcal isolates recovered from bulk-tank milk were biofilm-forming (*n* = 15, 57.7%) (Table S7). There was no statistical difference in the proportion of biofilm-forming isolates among *S. aureus* (77.8%) and that among non-*aureus* isolates (47.1%) (*p* = 0.13).

On 13 sampling occasions (27.1%), staphylococci similarly identified were isolated from samples of bulk-tank milk and samples of mammary secretion from animals in the farm.

### 3.4. Risk Factors Associated with High-Incidence Risk of Subclinical Mastitis

The results of the univariable analysis of correlations between incidence risk of subclinical mastitis/incidence risk of staphylococcal subclinical mastitis and the various parameters studied are detailed in Table S8.

**Figure 5:**
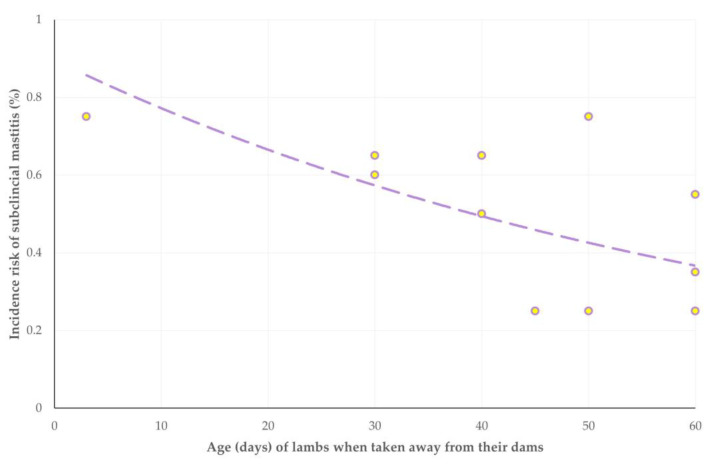
Scatterplot of incidence risk of subclinical mastitis and age when lambs were taken away from their dams in 12 sheep flocks in Greece monitored throughout a milking period (dashed line is trendline).

**Figure 6:**
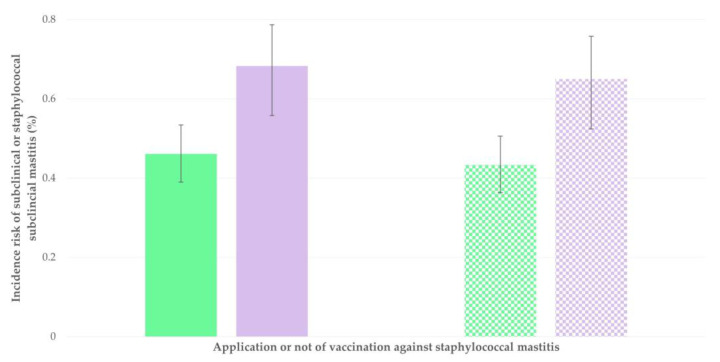
Incidence risk of subclinical mastitis (solid bars) or staphylococcal subclinical mastitis (motif bars) in sheep flocks in Greece monitored throughout a milking period, vaccinated (green bars) or not vaccinated (violet bars) against staphylococcal mastitis (bars indicate 95% confidence intervals).

With regard to subclinical mastitis, during the multivariable analysis, the following three variables emerged to be significantly associated (positively) with increased incidence risk of this infection: (a) younger age of newborns when taken away from the dam and consequently delayed start of milking of ewes (*p* = 0.025) (Figure 5), (b) omission of anti-staphylococcal mastitis vaccination of ewes (*p* = 0.042) (Figure 6) and (c) lack of employed staff on the farms (*p* = 0.046). It is also noted that there was a tendency for significance with lower level of education received by farmers (*p* = 0.055). Details are in Table 5.

With regard to staphylococcal subclinical mastitis, during the multivariable analysis, the following two variables emerged to be significantly associated (positively) with increased incidence risk of this infection: (a) younger age of newborns when taken away from the dam and consequently delayed start of milking of ewes (*p* = 0.008) and (b) omission of anti-staphylococcal mastitis vaccination of ewes (*p* = 0.017) (Figure 6). Details are in Table 6.

## 4. Discussion

### 4.1. Presence of Subclinical Mastitis in Flocks

There are differences in the protocols employed in field studies of ovine mastitis reported internationally, as these might have evaluated varying forms of the infection (clinical mastitis, subclinical mastitis or both) or might have included animals in differing production systems, i.e., dairy or meat-producing flocks; moreover, even among dairy flocks, the monitoring period may differ, i.e., beginning immediately after lambing or after the start of the milking period. All these reflect differences in the objectives of researchers, as well as in the available resources, but nevertheless make a direct comparison of the findings between studies difficult.

The finding regarding clinical mammary abnormalities (i.e., incidence risk: 1.3%) is to a large degree comparable to results reported previously by Lafi et al. [17] and Ruegg [18] in dairy ewes in Jordan and the United States and by Arsenault et al. [19] and Ridler et al. [20] in meat-producing sheep in Canada and New Zealand, respectively. However, it has not been possible to find in the international literature updated studies about the incidence of subclinical mastitis throughout a milking period. Nevertheless, the overall ‘positivity’ rate of 19.7% of samplings of animals in the study is comparable to 18.5% prevalence of subclinical mastitis found in Turkey [21] but smaller than 34.0% and 45.0% prevalence of subclinical mastitis found in Spain [22] and Italy [23].

It is of note that there were differences in our criteria for the definition of subclinical mastitis compared to those employed previously, by Las Heras et al. [22] and Dore et al. [23], who considered the presence of subclinical mastitis in cases of isolation of two or one, respectively, bacterial colonies on agar plates. These broader criteria have contributed to the higher prevalence rates reported in those studies compared to ours but have also reduced the specificity of the identification criteria.

The higher incidence risk of subclinical mastitis during the initial stage of the milking period may be the effect of the stress ensuing at the mammary glands as a result of the transition from lamb suckling to milking [4,24,25]. In contrast, the prevalence of the disease was found to increase, which occurs as the result of the recurrence of cases of the infection and thus accumulation of cases within flocks. The increased number of recurring cases of subclinical mastitis in flocks with higher incidence risk may possibly be associated with the higher number of pathogens circulating in such farms, which increase the chances of infections of animals throughout the milking period.

Subclinical mastitis exerts a marked adverse effect on the production of milk by affected ewes, which, in dairy sheep, is particularly important in terms of financial significance. This reduction in milk yield has been reported to amount to up to 55% [26]. It may occur in affected ewes independently of animal breed [4,27,28,29] and ultimately reflects into the cheese yield obtained from milk from affected animals [30].

### 4.2. Associations of Subclinical Mastitis with Milk Quality

#### 4.2.1. Protein Content in Bulk-Tank Milk

The composition of sheep milk has been traditionally considered to be primarily influenced by husbandry-related variables applied in farms, for example, breed and nutrition of animals or stage of lactation [30,31,32,33,34]. Hence, the chemical composition of sheep milk might be regulated by nutritional manipulations. For example, the protein content of milk can be altered by modifying the amount and the type of dietary protein provided to animals [31]. In general, however, the protein content of milk is less sensitive to nutritional manipulation than is fat content [35].

Recently, helminth infections (specifically, infection by *Teladorsagia* spp.) have been reported to be associated with reduced protein content in the bulk-tank milk of ewes [3]. This was attributed to the combined effects of a depressed appetite and a loss of plasma protein in parasitized animals. The larvae of these nematodes can invade the gastric glands in the abomasum and cause their destruction, and, that way, post-absorbance metabolism of proteins is impaired, leading to decreased protein content in milk [36,37]. In such cases, accompanying relevant clinical signs might also possibly be present in animals of the flock: suboptimal body condition and possibly also bodyweight loss.

The present findings also associated an increased prevalence of subclinical mastitis with a lower protein content in the bulk-tank milk of the farm. Hence, it may be suggested that perhaps control of infections (mastitis, parasitic infections) should be prioritized over husbandry-related interventions for the improvement of the composition of bulk-tank milk of a farm.

#### 4.2.2. Added Water in Bulk-Tank Milk

Subclinical mastitis leads to reduced milk production in affected ewes [38,39]. Moreover, in cases of increased prevalence of the infection in flocks, the somatic cell counts in the bulk tank also increase [40,41,42,43], as was also found in the present study. Hence, in such cases, the income of farmers would be reduced on two accounts: first, the reduced quantity of milk delivered to dairy companies and second, the reduced price of milk delivered as the result of the high somatic cell counts in there.

The positive correlation between the prevalence of subclinical mastitis and the added water in the bulk tank may possibly reflect the attempts of the farmers to compensate for the reduced income in such cases by adding water. Through this, the total quantity of milk delivered increased, leading to potential recuperation of the financial losses incurred because of subclinical mastitis. It should be noted that this finding certainly is not a direct consequence of the infection (i.e., subclinical mastitis) but rather an improper practice carried out by the farmers.

### 4.3. Identification of Risk Factors for Subclinical Mastitis

In sheep, as in other ruminant species, mastitis is a complex problem, characterized by a multifactorial etiology. Previous researchers have employed various approaches to study factors precipitating the infection, which have included experimental or observational studies [44], through which various variables have been identified to be involved in the process of mastitis development in ewes.

The present findings include differences between farms regarding the incidence risk of the infection: within-farm incidence risk varied from 25% to 75%. This large difference is consistent with the multifactorial nature of the infection.

Farms were assessed for a particularly large number of variables as potential risk factors for the infection; the number of variables evaluated is larger than in any other previous relevant study performed at the international level. Some of the variables found to be associated with higher-incidence risk of subclinical mastitis refer to health management practices followed in sheep farms, specifically the age of newborns at removal from the dam [45] and the application of anti-staphylococcal vaccination [5]. These factors relate to the potential for infection of the mammary glands and to the enhancement of the immune status of ewes and improving defenses against invading pathogens.

However, the emergence of variables related to the human resources in the flocks (presence of staff employed in the farms and level of education received by farmers) as being associated with the incidence of subclinical mastitis has not been reported previously. The availability of higher numbers of workforce in the farms (through the employment of outside workers) would contribute to the correct and timely performance of health management tasks, e.g., correct application of the milking routine and maintenance of the milking parlor, vaccination against mastitis, correct feeding of animals, etc. Further, the higher level of education among farmers in flocks where lower incidence risk of subclinical mastitis was recorded can be considered to be in line with the multifactorial facet of this infection; indeed, effective control of mastitis requires a variety of tasks, and higher levels of education improve cognitive functioning [46].

There are few studies available internationally on the possible effects of human resources in sheep farms on the health of the animals. In a study performed in New Zealand, it has been reported that older shepherds tended to omit essential health management tools, thus increasing the incidence of diseases in their farms [47]. In recent studies, it was reported that increased workforce on sheep farms has been associated with correct execution of a variety of tasks and positive production outcomes in these flocks [3] and that most predictors associated with the use of antibiotics in sheep farms were related to human resources rather than health management factors [3]. Moreover, Arce et al. [48] reported that the presence of women among the farming workforce led to decreased mastitis incidence in farms.

These findings should be taken into consideration, especially as the demographics of farmers in Europe gradually change and new entrants into agricultural business are of younger age, females, and with a higher educational background [49]. This pattern can also be associated with the trend for counter-urbanization and ‘return to the countryside’ prevalent in developed countries [50].

## 5. Conclusions

Given the significance of dairy sheep production in the agricultural sector of Greece [51], there is always interest in the study of mastitis and the monitoring of the situation in flocks in the country. The results have provided evidence regarding the situation in the country and have also identified factors that should be taken into account for controlling the infection. The findings also present another interaction between people and animals in the food-production chain, as well as the actions that farmers may take to recuperate income.

## Figures and Tables

**Table 1:** Frequency of isolation of bacteria from cases of subclinical mastitis among 12 sheep flocks in Greece monitored throughout a milking period.

Bacterial Identity	No. (Proportion) of Bacterial Isolates
*Corynebacterium* sp.	1 (0.5%)
*Escherichia coli*	3 (1.5%)
*Mannheimia haemolytica*	1 (0.5%)
*Micrococcus* sp.	1 (0.5%)
*Staphylococcus aureus*	38 (19.2%)
*Staphylococcus capitis*	2 (1.0%)
*Staphylococcus caprae*	6 (3.05)
*Staphylococcus chromogenes*	24 (11.1%)
*Staphylococcus epidermidis*	28 (15.2%)
*Staphylococcus equorum*	4 (2.0%)
*Staphylococcus haemolyticus*	4 (2.0%)
*Staphylococcus hominis*	5 (2.5%)
*Staphylococcus lentus*	9 (4.5%)
*Staphylococcus saprophyticus*	1 (0.5%)
*Staphylococcus schleiferi*	1 (0.5%)
*Staphylococcus sciuri*	3 (1.5%)
*Staphylococcus simulans*	34 (17.2%)
*Staphylococcus warneri*	2 (1.0%)
*Staphylococcus xylosus*	18 (9.1%)
*Streptococcus* spp.	13 (6.6%)
Total	198

**Table 2:** Mean (95% confidence interval) values of somatic cell counts and total bacterial counts in bulk-tank milk of 12 sheep flocks in Greece monitored during a milking period.

No. of Visit	Somatic Cell Counts (×10^6^ cells mL^−1^)	Total Bacterial Counts (×10^3^ cfu mL^−1^)
1st	0.391 (0.298–0.515) ^a,b^	232 (117–457) ^a^
2nd	0.390 (0.298–0.508) ^c,d^	165 (85–324) ^b^
3rd	0.671 (0.548–0.825) ^a,c^	184 (89–380) ^c^
4th	0.786 (0.634–0.974) ^b,d^	493 (275–871) ^a,b,c^

^a, b, c, d^: within the same column, differences between rows with identical superscripts are significant (*p* < 0.018); lack of superscripts indicates lack of significant difference between respective values.

**Table 3:** Mean (± standard error of the mean) values of chemical composition parameters in bulk-tank milk of 12 sheep flocks in Greece monitored during a milking period.

No. of Visit	Fat Content (%)	Total Protein Content (%)	Added Water (%)
1st	5.89 ± 0.23	4.52 ± 0.09	0.04 ± 0.06
2nd	6.00 ± 0.28	4.64 ± 0.07 ^a^	0.00 ± 0.00
3rd	5.89 ± 0.34	4.51 ± 0.06	0.58 ± 0.50
4th	5.89 ± 0.32	4.37 ± 0.05 ^a^	0.66 ± 0.45

^a^: within the same column, differences between rows with similar superscripts are significant (*p* < 0.035); lack of superscripts indicates lack of significant difference between respective values.

**Table 4:** Frequency of isolation of staphylococci from bulk-tank milk in 12 sheep flocks in Greece monitored during a milking period.

Bacterial Identity	No. (Proportion) of Bacterial Isolates
*Staphylococcus aureus*	9 (34.6%)
*Staphylococcus carnosus*	1 (3.8%)
*Staphylococcus chromogenes*	1 (3.8%)
*Staphylococcus equorum*	2 (7.7%)
*Staphylococcus haemolyticus*	2 (7.7%)
*Staphylococcus lugdunensis*	2 (7.7%)
*Staphylococcus pasteuri*	1 (3.8%)
*Staphylococcus simulans*	5 (19.2%)
*Staphylococcus xylosus*	3 (11.5%)
Total	26

**Table 5:** Results of multivariable analysis for variables with a significant association with increased incidence risk of subclinical mastitis.

Variables	Odds Risk (±se)/ Odds Ratios (95% CI) ^1^	*p*
Age (days) of newborns when taken away from the dam		0.037
Per day decrease	0.987 ± 1.004	0.037
Application of anti-staphylococcal vaccination		0.042
No (68.3% ^2^)	2.522 (1.360–4.678)	0.003
Yes (46.1%)	reference	-
Employment of working staff in the farm		0.046
No (60.6%)	3.022 (1.724–5.299)	0.0001
Yes (33.8%)	reference	-
Higher level of education received by farmer		0.055
Primary education (68.4%)	4.240 (2.185–8.228)	0.0001
Secondary or post-secondary education (53.8%)	2.281 (1.205–4.321)	0.011
Tertiary education (33.8%)	reference	-

^1^: se: standard error, CI: confidence interval; ^2^: incidence risk in farms where respective variable referred to.

**Table 6:** Results of multivariable analysis for variables with a significant association with increased incidence risk of staphylococcal subclinical mastitis.

Variables	Odds Risk (±se)/ Odds Ratios (95% CI) ^1^	*p*
Age (days) of newborns when taken away from the dam		0.007
Per day decrease	0.979 ± 1.002	0.007
Application of anti-staphylococcal vaccination		0.017
No (65.0% ^2^)	2.429 (1.324–4.456)	0.004
Yes (43.3%)	reference	-

^1^: se: standard error, CI: confidence interval; ^2^: incidence risk in farms where respective variable referred to.

## Data Availability

Most data presented in this study are in the Supplementary Materials. The remaining data are available on request from the corresponding author. The data are not publicly available as they form part of the PhD thesis of the first author, which has not yet been examined, approved and uploaded in the official depository of PhD theses from Greek universities.

## Supplementary Materials

The following supporting information can be downloaded at https://www.mdpi.com/article/10.3390/ani13203295/s1: Table S1: Details of 12 flocks included in a longitudinal study of subclinical mastitis; Table S2: Variables evaluated for potential association with incidence risk of subclinical mastitis in a longitudinal study throughout the milking period in 12 sheep flocks in Greece; Table S3: Details of multivariable models employed for the evaluation of predictors in a longitudinal study of subclinical mastitis among 12 sheep flocks in Greece monitored throughout a milking period; Table S4: Incidence risk of subclinical mastitis, incidence risk of staphylococcal subclinical mastitis and incidence risk of recurrence of subclinical mastitis among 12 sheep flocks in Greece monitored throughout a milking period; Table S5: Frequency of isolation of bacteria from cases of bacterial carriage in milk among 12 sheep flocks in Greece monitored throughout a milking period; Table S6: Results of biofilm formation by staphylococcal isolates from cases of subclinical mastitis or bacterial carriage in milk among 12 sheep flocks in Greece monitored throughout a milking period; Figure S1: Scatter-plot of prevalence of subclinical mastitis and total bacterial counts in bulk-tank milk in 12 sheep flocks in Greece monitored throughout a milking period; Table S7: Results of biofilm formation by staphylococcal isolates from bulk-tank milk among 12 sheep flocks in Greece monitored throughout a milking period; Table S8: Results of univariable analysis of variables evaluated for association with the outcomes ‘incidence risk of subclinical mastitis’ and ‘incidence risk of staphylococcal subclinical mastitis’ among 12 sheep flocks in Greece monitored throughout a milking period; Table S9: Published papers derived from PhD theses cited in the reference list of the main text, to which readers may refer for specific information regarding the citations in the text.
