# Supplementary material for: Longitudinal Study of Subclinical Mastitis in Sheep in Greece: An Investigation into Incidence Risk, Associations with Milk Quality and Risk Factors of the Infection
Source: Animals (Basel). 2023 Oct 22;13(20):3295. doi: 10.3390/ani13203295 (PMC10603708; doi:10.3390/ani13203295)
Supplement: Supplementary file 1 [file animals-13-03295-s001.zip › animals-2650409-supplementary.pdf]

**Table S1.** Details of 12 flocks included in a longitudinal study of subclinical mastitis.

| Farm | Location | Management system | No. of ewes | Breed of ewes | Start of lambing period | Annual milk production per ewe | Machine-milking | Milking sessions daily | Sampling period |
|---|---|---|---|---|---|---|---|---|---|
| 1 | Achaea, Western Greece | Semi-extensive | 130 | Friesian | September | 154 L | Yes | Two | 4 visits: November 2019 to June 2020 |
| 2 | Achaea, Western Greece | Semi-extensive | 170 | Friesian | October | 235 L | Yes | Two | 4 visits: December 2019 to June 2020 |
| 3 | Achaea, Western Greece | Semi-extensive | 180 | Local | September | 144 L | Yes | Two | 4 visits: November 2019 to June 2020 |
| 4 | Achaea, Western Greece | Intensive | 235 | Local | October | 111 L | Yes | Two | 4 visits: December 2019 to June 2020 |
| 5 | Achaea, Western Greece | Semi-intensive | 150 | Lacaune | September | 167 L | Yes | Two | 4 visits: November 2019 to June 2020 |
| 6 | Achaea, Western Greece | Semi-intensive | 230 | Chios | September | 296 L | Yes | Two | 4 visits: November 2019 to June 2020 |
| 7 | Corinthia, Peloponnese | Semi-intensive | 300 | Local | September | 293 L | Yes | Three | 4 visits: November 2019 to June 2020 |
| 8 | Corinthia, Peloponnese | Semi-intensive | 85 | Assaf | October | 400 L | Yes | Two | 4 visits: December 2019 to June 2020 |
| 9 | Corinthia, Peloponnese | Intensive | 600 | Chios | October | 183 L | Yes | Three | 4 visits: December 2019 to June 2020 |
| 10 | Corinthia, Peloponnese | Semi-intensive | 230 | Assaf | September | 357 L | Yes | Three | 4 visits: November 2019 to June 2020 |
| 11 | Corinthia, Peloponnese | Semi-intensive | 280 | Lacaune | September | 232 L | Yes | Two | 4 visits: November 2019 to June 2020 |
| 12 | Corinthia, Peloponnese | Semi-intensive | 145 | Local | October | 152 L | Yes | Two | 4 visits: November 2019 to June 2020 |

**Table S2.** Variables ($n$ = 67) evaluated for potential association with incidence risk of subclinical mastitis in a longitudinal study throughout the milking period in 12 sheep flocks in Greece.

| |
|---|
| Management system applied in the flocks (description according to EFSA classification) |
| Availability of a waiting area before the milking parlour (yes / no) |
| Material of the floor of the barn (cement / soil / slatted wood / slatted metal / other) |
| Availability of straw bedding (yes / no) |
| Annual frequency of removal / clean-up of the straw bedding (no. of occasions) |
| Grazing land available to animals (acres per animal) |
| No. of ewes in the flock (no.) |
| Breed of ewes (description) |
| Average age of culling females (years) |
| Month of the start of the lambing season (description) |
| Total milk quantity per ewe obtained during the preceding milking period (litres) |
| Average number of lambs born per ewe (no.) |
| Total visits made annually by veterinarians to the flock during the preceding season (no.) |
| Use of laboratory diagnostic examinations in samples of milk preventively (yes / no) |
| Age of lamb removal from their dams (days) |
| Daily number of milking sessions (no.) |
| Duration of the dry-period (months) |
| Administration of selenium to pregnant ewes (yes / no) |
| Administration of 'dry-ewe' treatment at the end of the lactation period (yes / no) |
| Use of teat disinfection after milking (yes / no) |
| Total number of antibiotics used for treatment of clinical mastitis (description, no.) |
| Route for administration of antimicrobials (systematically / intramammary) |
| Vaccination against mastitis (yes / no) |
| Annual frequency of systemic disinfections in the farm (no. of occasions) |
| Years since initial establishment or most recent renovation of the milking parlour (no.) |
| Volume of the parlour ($m^3$) |
| Material of the floor of the milking parlour (cement / tile / soil / other) |
| Type of milking parlour (fishbone / circular / linear parallel / linear one-sided / other) |
| Type of milking system (mobile / non-mobile) |
| Number of animal positions in the parlour (no.) |
| Number of available milking units per animal position (no.) |
| Provision of feed in the parlour (yes / no) |
| Availability of automated milk quantity measurement (yes / no) |
| Availability of milk quality indicators (yes / no) |
| Availability of milk flow indicators (yes / no) |
| System pulsation rate (p. $min^{-1}$) |
| System pressure (kPa.) |
| System pulsation rate to pressure ratio |
| Type of flow line (low / high / other) |
| Frequency of check-ups of the system by farmer (description) |
| Annual frequency of check-ups of the system by technicians (no. of occasions) |
| Water cleaning of parlour after the milking sessions (yes / no) |
| Temperature of cleaning water ($^{o}C$) |
| Use of detergent for parlour cleaning after the milking sessions (yes / no) |
| Frequency of changing teatcups (description) |
| Total number of wildlife species near the farm (description, no.) |
| Incidence of pregnancy toxaemia during the preceding year |

| |
|---|
| Incidence of lameness during the preceding lactation period |
| Incidence of obstetrical problems during the preceding lactation period |
| Number of occasions of administration of anthelmintic drugs annually (no.) |
| Duration of grazing during the year (no. of months) |
| Provision of hay as fodder to animals (yes / no) |
| Average quantity of hay provided daily to animals during the preceding season (kg) |
| Provision of straw to animals (yes / no) |
| Provision of silage to adult animals (yes / no) |
| Provision of finished feed (concentrate) to adult animals (yes / no) |
| Provision of finished feed (concentrate) to adult animals throughout the year (yes / no) |
| Type of finished feed (concentrate) provided to adult animals (description) |
| Average quantity of finished feed (concentrate) provided daily to animals during the preceding season (kg) |
| Age of the farmer (years) |
| Length of animal farming experience of the farmer (years) |
| Level of education of the farmer (description) |
| Farmer by profession (yes / no) |
| Daily period of presence in the farm (hours) |
| Family tradition in farming (yes / no) |
| Presence of working staff in the flock (yes / no) |

**Table S3.** Details of multivariable models employed for the evaluation of predictors in a longitudinal study of subclinical mastitis among 12 sheep flocks in Greece monitored throughout a milking period.

| Outcome | Variables Offered to The Multivariable Models ($n$) | Variables Required in the Final Models |
|---|---|---|
| Incidence risk of subclinical mastitis | 13 | (a) Age of lamb removal from their dams, (b) Administration of selenium to pregnant ewes, (c) Vaccination against mastitis, (d) Years sin-ce initial establishment or most recent renova-tion of the milking parlour, (e) Type of flow li-ne, (f) Frequency of changing teatcups, (g) Le-vel of education of the farmer, (h) Presence of working staff in the flock |
| Incidence risk of staphylococcalsubclinical mastitis | 10 | (a) Age of lamb removal from their dams, (b) Administration of selenium to pregnant ewes, (c) Vaccination against mastitis, (d) Years sin-ce initial establishment or most recent renova-tion of the milking parlour, (e) Frequency of changing teatcups, (f) Provision of finished fe-ed (concentrate) to adult animals throughout the year, (g) Average quantity of finished feed (concentrate) provided daily to animals dur- ing the preceding season, (h) Level of educa- tion of the farmer, (i) Presence of working staff in the flock |

**Table S4.** Incidence risk of subclinical mastitis, incidence risk of staphylococcal subclinical mastitis and recurrence risk of recurrence of subclinical mastitis among 12 sheep flocks in Greece monitored throughout a milking period.

| Farm | Incidence Risk of Subclinical Mastitis | Incidence Risk of Staphylo-coccal Subclinical Mastitis | Incidence Risk of Recurrence of Subclinical Mastitis |
|---|---|---|---|
| 1 | 50.0% | 50.0% | 23.1% |
| 2 | 65.0% | 65.0% | 42.9% |
| 3 | 35.0% | 35.0% | 28.6% |
| 4 | 25.0% | 25.0% | 33.3% |
| 5 | 25.0% | 25.0% | 20.0% |
| 6 | 75.0% | 60.0% | 25.0% |
| 7 | 55.0% | 50.0% | 45.5% |
| 8 | 25.0% | 25.0% | 44.4% |
| 9 | 60.0% | 55.0% | 42.9% |
| 10 | 65.0% | 55.0% | 75.0% |
| 11 | 75.0% | 75.0% | 22.2% |
| 12 | 65.0% | 65.0% | 44.4% |

**Table S5.** Frequency of isolation of bacteria from cases of bacterial carriage among 12 sheep flocks in Greece monitored throughout a milking period.

| Bacterial Identity | No. (proportion) of Bacterial Isolates |
|---|---|
| *Bacillus* spp. | 4 (2.3%) |
| *Corynebacterium* spp. | 2 (1.1%) |
| *Escherichia coli* | 2 (1.1%) |
| *Micrococuss* sp. | 1 (0.6%) |
| non-*aureus* staphylococci | 148 (84.1%) |
| *Streptococcus* spp. | 18 (10.2%) |
| *Trueperella* sp. | 1 (0.6%) |
| Total | 176 |

**Table S6.** Results of biofilm-formation by staphylococcal isolates from cases of subclinical mastitis or bacterial carriage among 12 sheep flocks in Greece monitored throughout a milking period.

| Bacterial Identity | No. of Bacterial Isolates | No. (Proportion) of Biofilm-forming Isolates |
|---|---|---|
| Isolates from cases of subclinical mastitis | | |
| *S. aureus* | 38 | 32 (84.2%) |
| *S. capitis* | 2 | 1 (50.0%) |
| *S. caprae* | 6 | 3 (50.0%) |
| *S. chromogenes* | 24 | 18 (75.0%) |
| *S. epidermidis* | 28 | 11 (39.3%) |
| *S. equorum* | 4 | 2 (50.0%) |
| *S. haemolyticus* | 4 | 2 (50.0%) |
| *S. hominis* | 5 | 4 (80.0%) |
| *S. lentus* | 9 | 3 (33.3%) |
| *S. saprophyticus* | 1 | 1 (100.0%) |
| *S. schleiferi* | 1 | 0 (0.0%) |
| *S. sciuri* | 3 | 1 (33.3%) |
| *S. simulans* | 34 | 26 (76.5%) |
| *S. warneri* | 2 | 1 (50.0%) |
| *S. xylosus* | 18 | 12 (66.7%) |
| Total | 179 | 117 (65.4%) |
| Isolates from cases of bacterial carriage | | |
| non-*aureus* staphylococci | 148 | 52 |
| Total | 148 | 52 (35.1%) |
| Grand total | 327 | 169 (51.7%) |

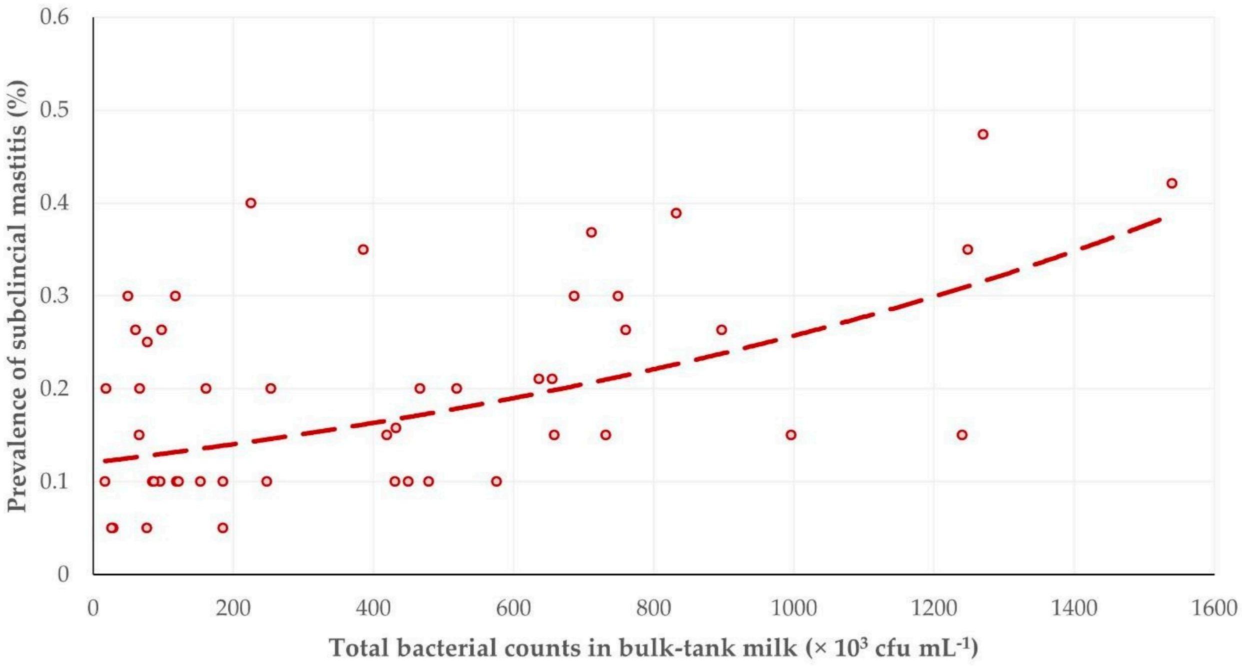


**Figure S1.** Scatter-plot of prevalence of subclinical mastitis and total bacterial counts in bulk-tank milk in 12 sheep flocks in Greece monitored throughout a milking period (dashed line is trendline).

**Table S7.** Results of biofilm-formation by staphylococcal isolates from bulk-tank milk among 12 sheep flocks in Greece monitored throughout a milking period.

| **Bacterial Identity** | **No. of Bacterial Isolates** | **No. (Proportion) of Biofilm-forming Isolates** |
|---|---|---|
| Isolates from cases of subclinical mastitis | | |
| *S. aureus* | 9 | 7 (77.8%) |
| *S. carnosus* | 1 | 0 (0.0%) |
| *S. chromogenes* | 1 | 1 (100.0%) |
| *S. equorum* | 2 | 0 (0.0%) |
| *S. haemolyticus* | 2 | 1 (100.0%) |
| *S. lugdunensis* | 2 | 0 (0.0%) |
| *S. pasteuri* | 1 | 1 (100.0%) |
| *S. simulans* | 5 | 4 (80.0%) |
| *S. xylosus* | 3 | 1 (33.3%) |
| Total | 26 | 15 (57.7%) |

**Table S8.** Results (Spearman's rank correlation coefficients) of univariable analysis of variables ($n$ = 67) evaluated for association with the outcomes 'incidence risk of subclinical mastitis' and 'incidence risk of staphylococcal subclinical mastitis' among 12 sheep flocks in Greece monitored throughout a milking period.

| | Subclinical Mastitis | | Staphylococcal Subclinical Mastitis | |
|---|---|---|---|---|
| **Variables** | **Correlation Coefficient ($r_{sp}$)** | ***p*-Value** | **Correlation Coefficient ($r_{sp}$)** | ***p*-Value** |
| Management system applied in the flocks | –0.078 | 0.81 | –0.016 | 0.96 |
| Availability of a waiting area before the milking parlour | n/r | n/r | n/r | n/r |
| Material of the floor of the barn | n/r | n/r | n/r | n/r |
| Availability of straw bedding | –0.399 | <0.20 | –0.398 | >0.20 |
| Annual frequency of removal / clean-up of the straw bedding | 0.313 | 0.32 | 0.182 | 0.57 |
| Grazing land available to animals | –0.336 | 0.29 | –0.305 | 0.34 |
| No. of ewes in the flock | 0.248 | 0.44 | 0.195 | 0.54 |
| Breed of ewes | 0.015 | 0.96 | –0.131 | 0.69 |
| Average age of culling females | –0.340 | 0.28 | –0.333 | 0.29 |
| Month of the start of the lambing season | 0.165 | 0.61 | 0.242 | 0.45 |
| Total milk quantity per ewe obtained during the preceding milking period | 0.284 | 0.37 | 0.145 | 0.65 |
| Average number of lambs born per ewe | 0.005 | 0.99 | 0.170 | 0.60 |
| Total visits made annually by veterinarians to the flock during the preceding season | 0.061 | 0.85 | 0.045 | 0.89 |
| Use of laboratory diagnostic examinations in samples of milk preventively | 0.149 | 0.64 | –0.050 | 0.88 |
| Age of lamb removal from their dams | –0.577 | 0.049 | –0.656 | 0.021 |
| Daily number of milking sessions | 0.170 | 0.6 | 0.113 | 0.73 |
| Duration of the dry-period | –0.035 | 0.91 | 0.012 | 0.97 |
| Administration of selenium to pregnant ewe | 0.494 | 0.10 | 0.426 | 0.17 |
| Administration of 'dry-ewe' treatment at the end of the lactation period | 0.049 | 0.88 | 0.147 | 0.65 |
| Use of teat disinfection after milking | –0.296 | 0.35 | –0.295 | 0.35 |
| Antibiotics used for treatment of clinical mastitis | –0.110 | 0.73 | –0.115 | 0.72 |
| Route for administration of antimicrobials | 0.115 | 0.72 | 0.044 | 0.89 |
| Vaccination against mastitis | 0.567 | 0.05 | 0.593 | 0.042 |
| Annual frequency of systemic disinfections in the farm | –0.135 | 0.68 | –0.177 | 0.58 |
| Years since initial establishment or most recent renovation of the milking parlour | –0.539 | 0.07 | –0.436 | 0.16 |
| Volume of the parlour | 0.299 | 0.35 | 0.204 | 0.53 |
| Material of the floor of the milking parlour | 0.177 | 0.58 | 0.135 | 0.68 |
| Type of milking parlour | n/r | n/r | n/r | n/r |
| Type of milking system | –0.399 | < 0.20 | –0.398 | > 0.20 |
| Number of animal positions in the parlour | 0.261 | 0.41 | 0.214 | 0.50 |
| Number of available milking units per animal position | 0.319 | 0.27 | 0.305 | 0.33 |
| Provision of feed in the parlour | 0.057 | 0.86 | –0.056 | 0.86 |
| Availability of automated milk quantity measurement | n/r | n/r | n/r | n/r |
| Availability of milk quality indicators | n/r | n/r | n/r | n/r |
| Availability of milk flow indicators | –0.222 | 0.49 | –0.088 | 0.78 |
| System pulsation rate | –0.468 | 0.12 | –0.389 | 0.21 |
| System pressure | –0.268 | 0.40 | –0.237 | 0.46 |
| System pulsation rate to pressure ratio | –0.073 | 0.82 | 0.010 | 0.98 |
| Type of flow line | 0.708 | 0.010 | 0.735 | 0.006 |

| | | | | |
|---|---|---|---|---|
| Frequency of check-ups of the system by farmer | 0.112 | 0.73 | 0.261 | 0.41 |
| Annual frequency of check-ups of the system by technicians | –0.163 | 0.61 | –0.143 | 0.66 |
| Water cleaning of parlour after the milking sessions | n/r | n/r | n/r | n/r |
| Temperature of cleaning water | –0.011 | 0.97 | –0.091 | 0.79 |
| Use of detergent for parlour cleaning after the milking sessions | n/r | n/r | n/r | n/r |
| Frequency of changing teatcups | 0.470 | 0.12 | 0.579 | 0.049 |
| Total number of wildlife species near the farm | –0.340 | 0.28 | –0.266 | 0.40 |
| Annual incidence of pregnancy toxaemia during the preceding lactation period | –0.269 | 0.40 | –0.170 | 0.60 |
| Annual incidence of lameness during the preceding lactation period | –0.197 | 0.54 | –0.284 | 0.37 |
| Annual incidence of obstetrical problems during the preceding lactation period | –0.452 | 0.14 | –0.363 | 0.25 |
| Number of occasions of administration of anthelmintic drugs annually | –0.107 | 0.74 | –0.046 | 0.89 |
| Duration of grazing during the year | –0.195 | 0.54 | –0.195 | 0.54 |
| Provision of hay as fodder to animals | n/r | n/r | n/r | n/r |
| Average quantity of hay provided daily to animals during the preceding season | 0.156 | 0.63 | 0.029 | 0.93 |
| Provision of straw to animals | –0.395 | > 0.20 | –0.361 | 0.25 |
| Provision of silage to adult animals | –0.028 | 0.93 | –0.113 | 0.73 |
| Provision of finished feed (concentrate) to adult animals | n/r | n/r | n/r | n/r |
| Provision of finished feed (concentrate) to adult animals throughout the year | 0.567 | 0.05 | 0.762 | 0.004 |
| Type of finished feed (concentrate) provided to adult animals | –0.186 | 0.56 | –0.343 | 0.28 |
| Average quantity of finished feed (concentrate) provided daily to animals during the preceding season | 0.392 | 0.21 | 0.416 | 0.18 |
| Age of the farmer | –0.173 | 0.59 | –0.179 | 0.58 |
| Length of animal farming experience of the farmer | –0.305 | 0.34 | –0.185 | 0.57 |
| Level of education of the farmer | –0.766 | 0.004 | –0.718 | 0.008 |
| Farmer by profession | 0.222 | 0.49 | 0.354 | 0.26 |
| Daily period of presence in the farm | –0.032 | 0.92 | –0.029 | 0.93 |
| Family tradition in farming | 0.296 | 0.35 | 0.295 | 0.35 |
| Presence of working staff in the flock | 0.676 | 0.016 | 0.648 | 0.023 |

**Table S9.** Published papers derived from PhD theses cited in the reference list of the main text, to which readers may refer for specific information regarding the citations in the text.

| PhD Theses Cited in the Reference List of the Main Text | Published Papers Derived from These Theses |
| --- | --- |
| Lianou, D.T. *Mapping the Small Ruminant Industry in Greece: Health Management and Diseases of Animals, Preventive Veterinary Medicine and Therapeutics, Reproductive Performance,Production Outcomes, Veterinary Public Health, Socio-demographic Characteristics of the Farmers*. PhD thesis, University ofThessaly, Volos, Greece, 2023. | -D.T. Lianou et al. (2020). A detailed questionnaire for the evaluation of health management in dairy sheep and goats. *Animals*, 10:1489.<br>-D.T. Lianou, G.C. Fthenakis (2021). Dairy sheep and goat farmers: socio- demographic characteristics and their associations with health management and performance on farms. *Land*, 12:1358.<br>-D.T. Lianou et al. (2021). Extensive countrywide field investigation of somatic cell counts and total bacterial counts in bulk-tank raw milk insheep flocks in Greece. *Foods*, 10:268.<br>-D.T. Lianou, G.C. Fthenakis (2022). Use of antibiotics against bacterial infections on dairy sheep and goat farms: patterns of usage and associations with health management and human resources. *Antibiotics*, 11:753.<br>-D.T. Lianou et al. (2023). Data on mapping 444 dairy small ruminant farms during a countrywide investigation performed in Greece. *Animals*, 13:2044 |
| Vasileiou, N.G.C. *Mastitis in Ewes Associated with Staphylococcus spp.: New Clinical,Epidemiological, Management, Microbiological, and Zoonoticfindings and Evaluation of a NovelVaccine Against the Disease*. PhD thesis, University of Thessaly,Volos, Greece, 2019 | -N.G.C. Vasileiou et al. (2018) Extensive countrywide field investigation of subclinical mastitis in sheep in Greece. *Journal of Dairy Science*, 101:7297–7310.<br>-N.G.C. Vasileiou et al. (2018) Slime-producing staphylococci as causal agents of subclinical mastitis in sheep. *Veterinary Microbiology*, 224:93–99.<br>-N.G.C. Vasileiou et al. (2019) Evaluation of efficacy of a biofilm-embeddedbacteria-based vaccine against staphylococcal mastitis in sheep - a randomized, placebo-controlled field study. *Journal of Dairy Science*, 102:9328-9344.<br>-N.G.C. Vasileiou et al. (2019) Experimental study for evaluation of the efficacy of a biofilm-embedded bacteria-based vaccine against *Staphylococcus chromogenes*-associated mastitis in sheep. *Veterinary Microbiology*, 239:108480. |
